# In silico and computational analysis of zinc finger motif-associated homeodomain (ZF-HD) family genes in chilli (Capsicum annuum L)

**DOI:** 10.1186/s12864-023-09682-x

**Published:** 2023-10-11

**Authors:** Md. Abir Ul Islam, Juthy Abedin Nupur, Muhammad Shafiq, Qurban Ali, Adnan Sami, Muhammad Adnan Shahid

**Affiliations:** 1 United Graduate School of Agricultural Science, Faculty of Biological Sciences, Gifu University, Yanagido, Gifu, 501-1193 Japan; 2 Department of Agricultural, Food and Nutritional Science, University of Alberta, Edmonton, AB T6G 2R3 Canada; 3 Department of Horticulture, University of Panjab, Lahore, 54000 Pakistan; 4 Department of Plant Breeding and Genetics, Faculty of Agricultural Sciences, University of the Punjab, P.O BOX. 54590, Lahore, Pakistan; 5 Horticultural Science Department, North Florida Research and Education Center, University of Florida/IFAS, Quincy, FL USA

**Keywords:** Zinc Finger-HD (ZHD), Transcription factor, *Capsicum annuum*, In silico analysis

## Abstract

Zinc finger-homeodomain (ZHD) proteins are mostly expressed in plants and are involved in proper growth and development and minimizing biotic and abiotic stress. A recent study identified and characterized the ZHD gene family in chilli (*Capsicum annuum* L.) to determine their probable molecular function. ZHD genes with various physicochemical characteristics were discovered on twelve chromosomes in chilli. We separated ZHD proteins into two major groups using sequence alignment and phylogenetic analysis. These groups differ in gene structure, motif distribution, and a conserved ZHD and micro-zinc finger ZF domain. The majority of the CaZHDs genes are preserved, early duplication occurred recently, and significant pure selection took place throughout evolution, according to evolutionary study. According to expression profiling, the genes were found to be equally expressed in tissues above the ground, contribute to plant growth and development and provide tolerance to biotic and abiotic stress. This in silico analysis, taken as a whole, hypothesized that these genes perform distinct roles in molecular and phytohormone signaling processes, which may serve as a foundation for subsequent research into the roles of these genes in other crops.

**Supplementary Information:**

The online version contains supplementary material available at 10.1186/s12864-023-09682-x.

## Introduction

*Capsicum annuum* L., commonly known as chilli pepper, is a highly diverse species cultivated and traded globally for centuries. In our recent genome-wide analysis, we performed an in silico characterization of the Zinc Finger-HD (ZF-HD) transcription factor gene family in *Capsicum annuum* L [1]. Chili peppers are known for their pungency, which is due to the presence of capsaicinoids, a group of secondary metabolites that provide protection against herbivores and pathogens [2]. Our analysis revealed that the ZF-HD transcription factor gene family in *Capsicum annuum* L. includes multiple members with varying degrees of conservation across different cultivars [3]. These findings have important implications for improving our understanding of the molecular mechanisms underlying the diverse phenotypic traits observed in chilli pepper cultivars, including heat, color, and shape. In addition to their culinary uses, chilli peppers have also been traditionally used for their anti-inflammatory, analgesic, and antioxidant properties [4]. Plant's physiological activities and expression regulation are strongly linked to Transcription Factors [5], which control growth, development, and stress response [6]. The DNA-binding domains of each TF family are distinct, forming their binding specificity. A group of transcription factors is generated through zinc finger-homeodomain (ZHD) genes which are found to contribute differently to plants' growth and development [7]. It has been discovered that ZHD genes are connected with photosynthesis, defense mechanism, and various stress in plants [4–6]. ZHD proteins are expressed in floral tissue in advance or, more specifically, indicating that they can regulate flowering in plants [8]. Different regulatory proteins contain zinc finger motifs. These motifs are composed of two pairs of conserved cysteine or histidine residues which provide consistency to the motifs into a finger-shaped loop vis coordinating with a single zinc ion [9].

Furthermore, a single protein can carry one or more zinc finger motifs, mainly contributing to DNA-binding protein–protein interaction [10]. The zinc finger-homeodomain (ZHD) gene family members were first discovered in the C4 plant species *Flaveria* [11]. Previously ZF-HD1 was identified in *Arabidopsis* to observe its function, where it was found that ZF-HD1 binds with the EARLY RESPONSE TO DEHYDRATION STRESS 1 (ERD1) promoter due to induction of salt, increasing transpiration rate as well as abscisic acid [12]. Moreover, when this ZF-HD1 overexpressed and joined with NAC genes, it assists in developing drought tolerance in plants [12]. In certain crops, such as rice the members of the ZF-HD group have been identified. Specifically, researchers have discovered fourteen zinc finger homeobox genes in rice that belong to the ZF-HD group [13]. After conducting in-depth evolutionary analysis, which included examining the phylogenetic tree, duplication events, and comparative analysis, researchers could identify ancestral relationships between the ZF-HD gene and other genes. This analysis revealed how the ZF-HD gene had evolved and diversified over time, providing insights into its functional and structural properties For instance, MIF genes (Macrophage Migration Inhibitory Factor) exhibit only zinc finger while ZF-HD has both zinc finger as well as homeodomain, which indicates that MIF genes might have been produced from ZF-HD through many evolutionary changes or ZF-HD originated from MIF through gaining homeodomain portion [14].

ZHD-regulated transcription factors have been identified and studied in hot pepper (*Capsicum annuum* L.), contributing to our understanding of their function in plant growth, development, and stress response. Therefore, this in silico paper described the evolutionary trend, protein–protein interaction, and expression pattern of ZHD TFs in chilli under optimal and adverse conditions and explained the possible mechanisms of ZHD transcription factors in chilli plants.

## Materials and methods

### Sequence retrieval from the database

Hidden Markov Model (HMM) based conserved domain of ZHD gene family (PF) firstly downloaded from the Pfam database (Pfam 35.0: http://pfam.xfam.org/). The conserved domain HMM profile was used for BLASTP search against Sol Genomic (https://solgenomics.net/organism/Capsicum_annuum/genome) and China National Gene Bank (https://db.cngb.org/) databases for the release 2.0 cv. Zunla-1 genome with an expected cut-off value of 0.01 [15]. SMART (http://smart.embl-heidelberg.de/) and NCBI CDD (Conserved Domain Database) (https://www.ncbi.nlm.nih.gov/Structure/cdd/wrpsb.cgi) were used to analyze the simple molecular architecture of the retrieved predicted amino acid sequences to find out either the sequences possessed the PF domain or not. Those which did not contain the required conserved domain were excluded [12, 13].

### Physiochemical properties determination

ProtParam tool was used to retrieve some physiochemical properties like protein length (amino acid residues), molecular weight, isoelectric point [16], and GRVY, while the subcellular localization was found in the Cell-Ploc-20 [17]. Gene IDs, chromosomal positions, direction, and sequences of proteins and CDS of the potential genes were collected from Sol Genomics, but genomic sequences were retrieved from the NCBI (https://www.ncbi.nlm.nih.gov/). All those predicted Chilli’s ZHD family genes were renamed following their chromosomal locations.

### Conserved motif analysis, domain prediction, and exon–intron distribution

The CaZHD gene family proteins’ motifs analysis was done with the help of MEME suite (https://meme-suite.org/meme/) [18] with the default conditions included motifs 20, minimum width 6, and maximum width 50. The hit data from the NCBI CDD, Norwich phytogenic data from MEGA 11.0 and MEME suite data were used in TBtools to visualize the conserved domains and motifs (TB) [19]. The genomic and CDS sequences of the CaZHD gene family are used in the gene structure display server (GSDS) web tool to analyze their exon and intron distribution [20].

### Multiple sequence analysis and phylogenetic tree analysis

Crops like Chilli (*Capsicum annuum*) 11 and *Arabidopsis* (15) amino acid sequences of ZHD family were aligned with the help of ClustalX v2.1 multiple sequence alignment tool [21], and then for colorful visualization, the aligned data exported to Genedoc (https://www.nrbcs.org/gfx/genedoc/ebinet.htm) [22]. ZHD protein sequences of Arabidopsis, Maize, Tomato and chilli were used to build phylogenetic tree using the neighbor-joining (NJ) technique with 1000 bootstrap replicates with the help of MEGA 11.0 software [23] and representation of the tree updated using iTOL (https://itol.embl.de/) [24].

### Cis‑regulatory elements analysis and function determination

The 1500 upstream promotor regions were extracted from NCBI (Link) for cis-regulatory elements extraction as they bind to the transcription factors and regulate target gene functions [25]. The PlantCARE (http://bioinformatics.psb.ugent.be/webtools/plantcare/html/) web tool retrieves 5 to 20 bp putative cis-elements for the promotor region [26]. The measured cis-regulatory elements results are visualized in a heatmap with the help of TBtools [19].

### Gene duplication and synteny analysis

A NCBI BLASTP search was done among the ZHD gene protein sequences of the chilli using 80% sequence identity for determining gene duplication [27]. The synonymous substitution rate (Ks), nonsynonymous substitution rate (Ka), and Ka/Ks ratio among the duplicated gene pairs were calculated with the help of KaKs calculator 2.0 [28]. A well-established formula T = Ks/2λ (where λ = 6.5 × 10− 9) was used to measure the evolutionary divergence. Gene duplication events among the chilli-*Arabidopsis*, chilli-tomato, and chilli-maize were analyzed using the Multiple Collinearity Scan toolkit (MCScanX) [29]. To exhibit the syntenic relationship of the ZHD genes of Chilli, Micro Synteny view software in TBtools was used to construct a map [19].

### Gene ontogeny analysis

The ShinyGO v0.75: Gene Ontology Enrichment Analysis + more (http://bioinformatics.sdstate.edu/go/) was used to obtain gene ontology (GO) annotation against *Capsicum annuum* where potential chilli candidate proteins and/or genes IDs were subjected. The *p*-value cut-off (FDR) at 0.01 is set to calculate GO enrichment.

### Transcriptome analysis

The expression profile of Zulna-1 for different parts such as fruit, flower, leaf, meristem, root and stem were extracted from the NCBI GEO database to analyze the organ-specific expression profile of CaZHD gene family (https://www.ncbi.nlm.nih.gov/geo/) [30]. For expression profiling, RNA-seq data unit values Read Per Kilobases per Million mapped reads (RPKM) were log2 folded, transformed and hierarchically displayed the heatmap through Heatmap Illustrator in TBtools [19].

### qRT-PCR analysis for Investigating CaZHD genes in root

The samples were subjected to RNA isolation using RNA-easy Isolation Reagent from Vazyme, Nanjing, China. The quality of the isolated RNA was assessed using 0.8% agarose gel electrophoresis, while the purity and concentration were determined using a NanoDrop 2000 Spectrophotometer. Samples with OD_260_/OD_280_ ratios ranging from 1.90 to 2.10 were considered suitable for further experiments. For the analysis of CaZHD genes, the RNA samples were reverse-transcribed into cDNA using Hifair™ II 1st Strand cDNA Synthesis SuperMix for qPCR. The subsequent qRT-PCR analyses were carried out on a Light Cycler 480 instrument with a 20-μL reaction mixture (Primers Table S1). The Livak method was employed to calculate the relative levels of gene transcripts. Each RT-qPCR analysis was performed using three biological replicates. Protein-Protein interaction analysis.

With a high confidence score of 0.7, Protein-Protein Interaction (PPI) analysis was performed using STRING v11.0 (https://string-db.org/) [31]. The interactome's functional enrichment analysis was done somewhere at 0.01 level. The PPI network was created using active interaction based on various sources, including text mining, experiments, gene fusion, databases, co-expression, and an interaction score > 0.4. The physical and functional roles of the major candidate genes implicated were determined using this interactome map.

### Putative microRNA target site analysis 

The first mature miRNA from the PmiREM server (https://www.pmiren.com/) was used to determine the target site of chilli's 11 CaZHD gene family. The CDS of the 11 genes was then compared to the mature miRNA using the PsRNA online server tool (https://www.zhaolab.org/psRNATarget/) using the default setting [32]. Using the Cytoscape program (https://www.omicshare.com/tools/), a connection between the predicted miRNA was created [29, 30].

## Results

### Identification and physicochemical property of *CaZHD *TF gene family

**Fig. 1:**
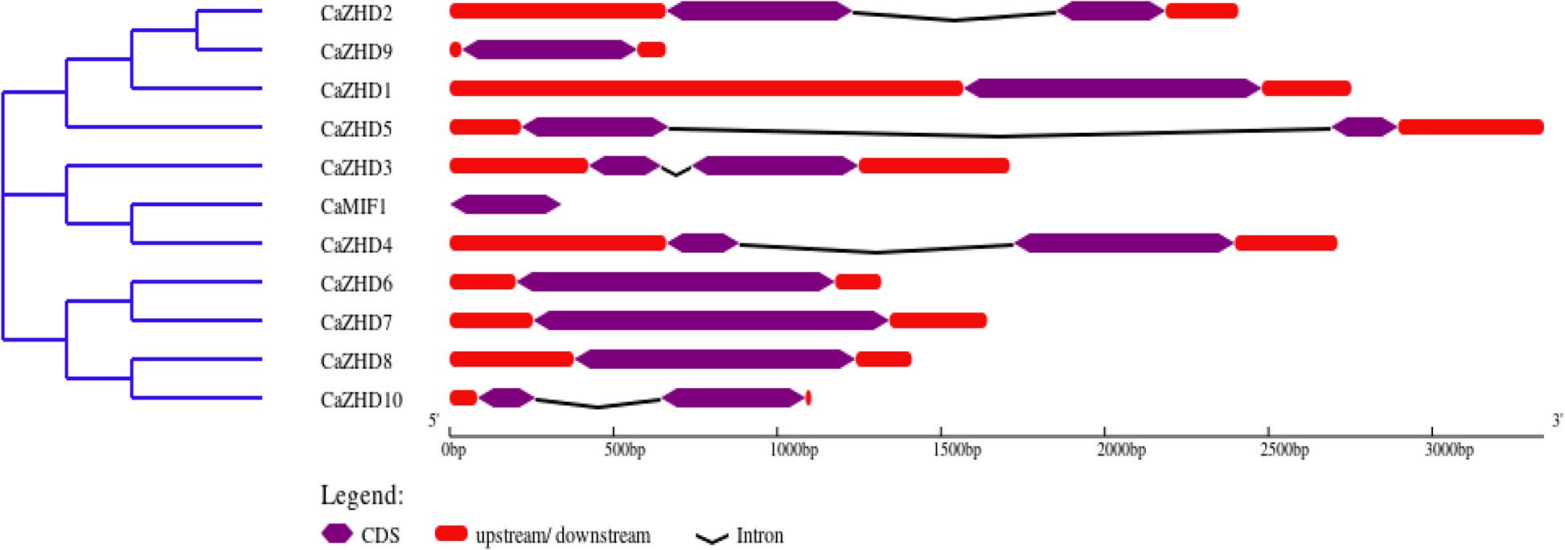
Phylogenetic relationships and gene structures of the ZHD genes from chilli. **A** The phylogenetic tree was constructed based on the full-length sequences of *CaZHD* genes. **B** Intron–Exon structures of the *CaZHD* genes. Purple boxes indicate exons, red boxes UTR region, and black lines indicate introns

A total of 11 ZF-HD genes were identified from the Sol Genomics, consisting of two conserved domains, the ZF domain and Micro Zinc Finger ZF domain (Fig. 1). All the 11 ZHD genes of chilli were named *CaZHD1-CaZHD10,* and micro zinc finger *CaMIF1* was designated based on their corresponding location on the chromosomes 1-12 top to bottom. The length of the genes ranged from 125 -362aa, where *CaMIF1* and *CaZHD7* have the lowest and highest values, respectively (Table 1). *CaMIF* cellular location was either the nucleus or chloroplast, while the remaining CaZHDs were localized in the nucleus (Table 1). The *CaZHDs* were found to be more conserved as only five among eleven have only one intron (Fig. 1).

**Table 1:** Information of eleven (11) ZHD genes of chilli (*Capsicum* *annuum*)

Gene name	Accession Number		Gene location			Direction	No. of Amino Acids	pI-value	GRAVVY	Molecular weight (KD)	Subcellular location
	Gene ID	Protein ID	Chromosome Number	Start	End						
*CaMIF1*	Capana08g001879	KAF3648334.1	PGAv.1.6scaffold798	648,227	648,568	R	**125**	9.14	-0.149	**12,776.77**	**Chloroplast. Nucleus**
*CaZHD1*	Capana02g002717	XP_01656064	2	149,557,535	149,560,287	F	303	8.15	-1.138	34,418.18	Nucleus
*CaZHD2*	Capana04g000340	XP_016568547	4	5,400,760	5,403,167	R	301	8.69	-0.9	32,899.57	Nucleus
*CaZHD3*	Capana04g000918	XP_016571526	4	21,394,574	21,396,283	F	244	**7.09**	-1.014	27,325.25	Nucleus
*CaZHD4*	Capana08g001767	XP_016538942	8	136,187,812	136,190,522	F	299	7.8	-0.834	33,659.17	Nucleus
*CaZHD5*	Capana05g000239	XP_016574518	5	3,902,151	3,905,491	F	241	8.67	-0.838	24,229.21	Nucleus
*CaZHD6*	Capana02g000908	XP_016559049.1	2	101,840,237	101,841,554	F	324	8.84	-0.82	35,645.78	Nucleus
*CaZHD7*	Capana02g000909	XP_016559052.1	2	101,969,817	101,971,457	R	**362**	8.62	-0.897	**39,682.94**	Nucleus
*CaZHD8*	Capana05g001601	XP_016574674.1	5	156,124,439	156,125,849	R	286	8.51	-0.969	31,767.23	Nucleus
*CaZHD9*	Capana03g001589	XP_016567717.1	3	30,305,471	30,306,130	F	178	8.41	-0.828	19,847.93	Nucleus
*CaZHD10*	Capana08g001884	XP_016582292.1	8	137,789,911	137,791,014	R	221	**9.35**	-0.601	23,104.26	Nucleus

### Conserved domain and gene structure analysis of ZHD 

**Fig. 2:**
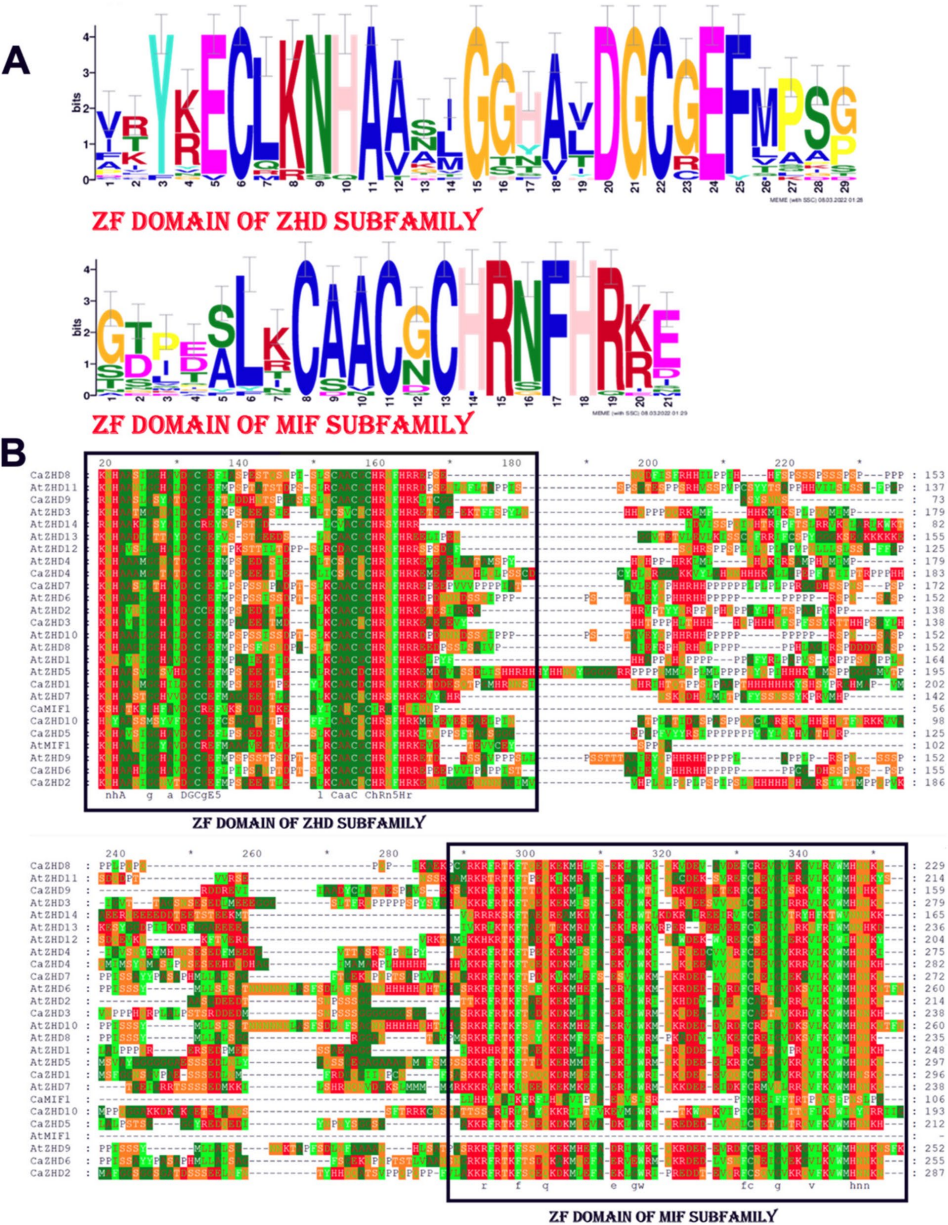
The sequence logos are based on alignments of *CaZHD* domains (**A**). *ZF* domain of ZHD subfamily and MIF subfamily is highly conserved across all *ZHD* proteins in Chilli. Multiple alignment analysis of *CaZHD* domains was performed with MUSCLE (**B**)

**Fig. 3:**
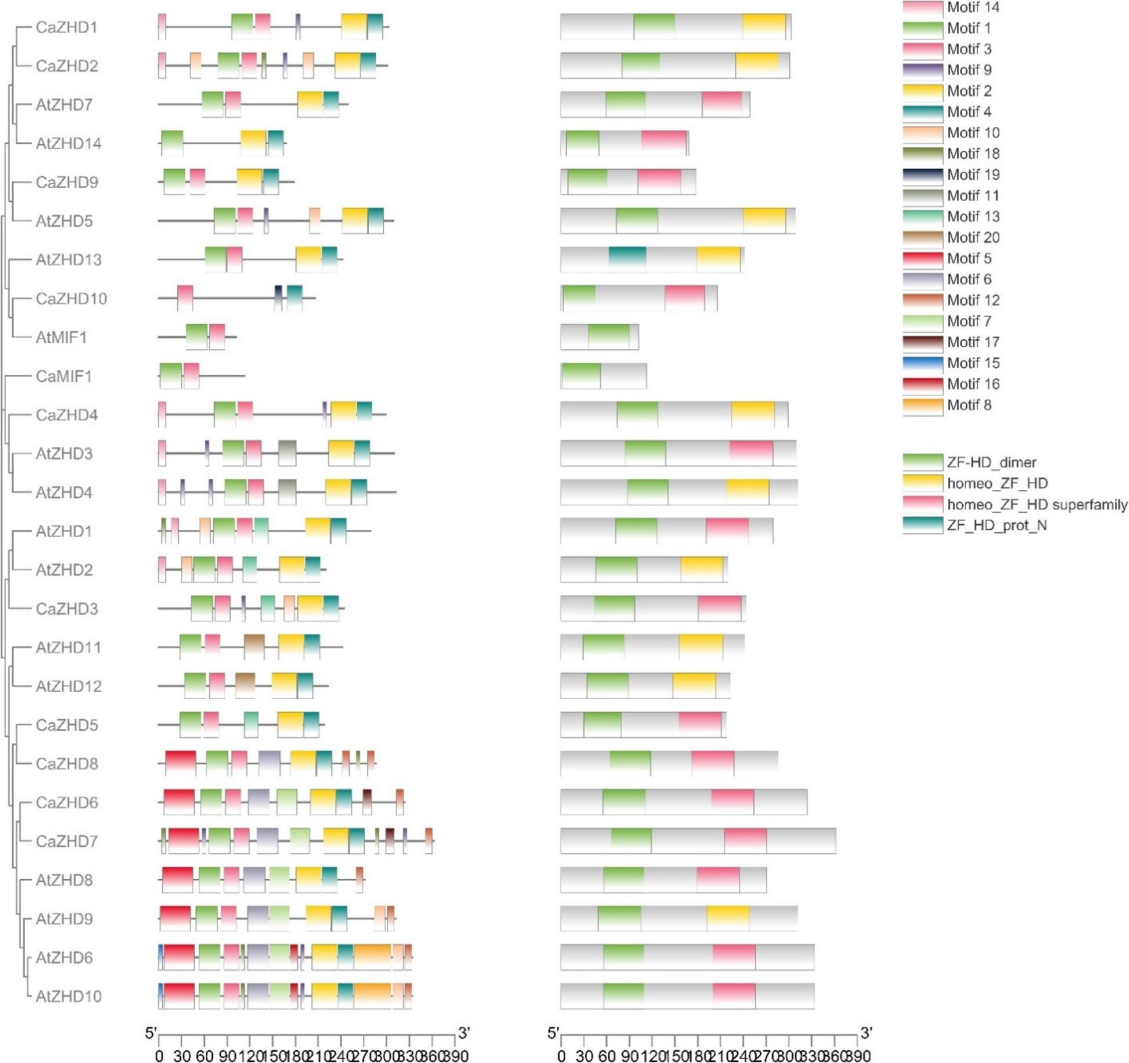
The distribution of 20 motifs on *ZHD* proteins of Arabidopsis and chilli by using MEME version 4.9.0 and interlinking it with a phylogenetic tree to better understand their association. The bars represent motifs with different color codes for different types of motifs

The conserved domain analysis showed that all the ZHD genes of Arabidopsis and chilli had two completely conserved domains: ZF Domain of ZHD subfamily (Fig. 2) and the ZF Domain of MIF subfamily (Fig. 2). Twenty conserved motifs in the ZHD gene family were identified using MEME online tools in Arabidopsis and chilli (Fig. 3, Supplementary Table 1). The results showed that most of genes of the ZHD family had four typical motifs, motif 1 (ZF-HD_dimer), motif 2 (Homeo_ZF_HD), motif 3 (Homeo_ZF_HD superfamily), and motif 4 (ZF-HD_prot_N), indicating that these four motifs were relatively conserved among the ZHD gene family (Fig. 3). Motif 1 and motif 3 were present in most of the ZHD genes of Arabidopsis and chilli except *CaZHD10, AtZHD14,* respectively. Moreover, motif 2 was not present in *CaZHD10*, *AtMIF1* and *CaMIF1,* while Arabidopsis and chilli MIF do not contain motif4.

### Phylogenetic analysis and classification of the ZHD transcription factor family

**Fig. 4:**
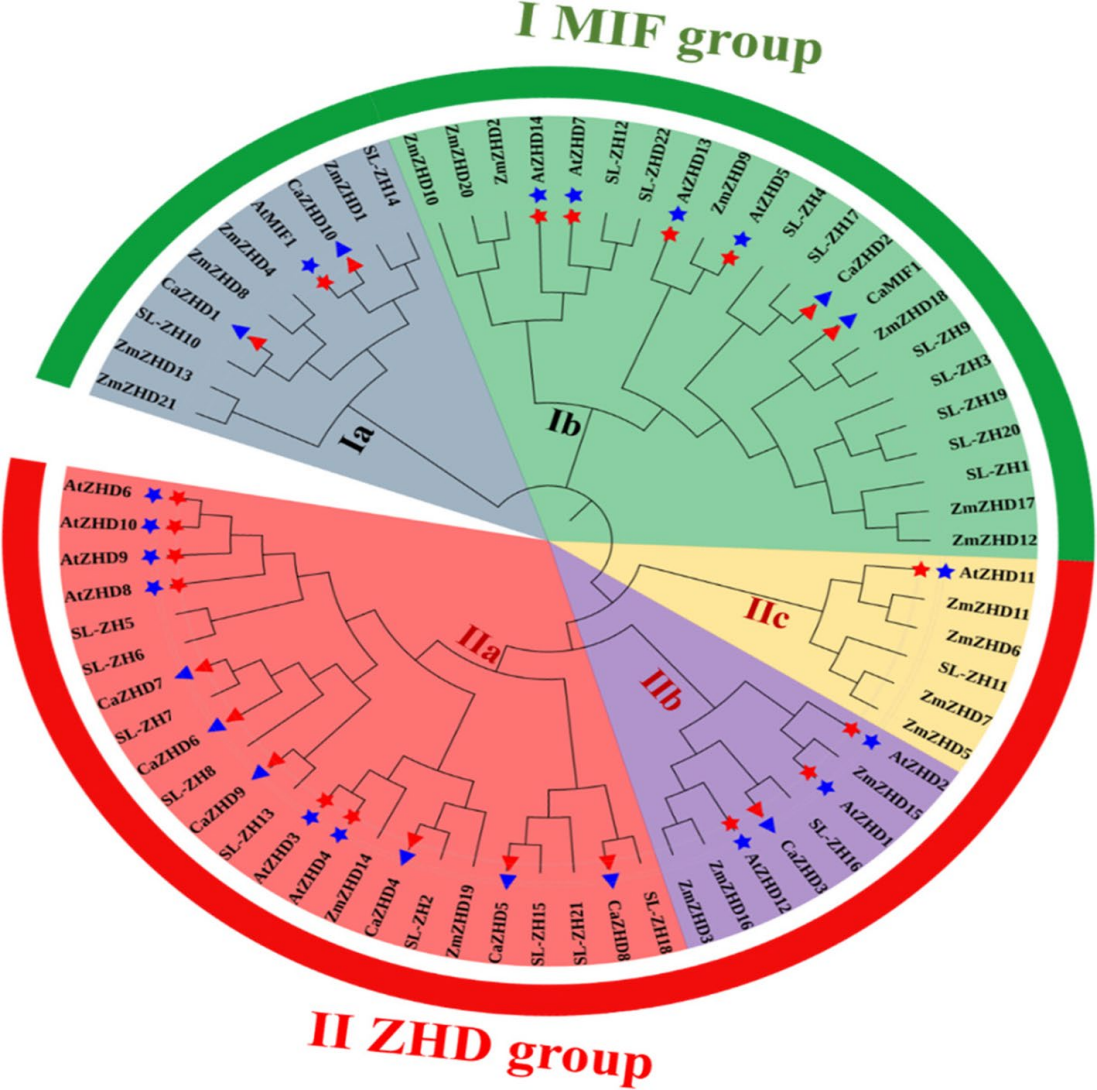
Phylogenetic Relationship among *ZHD* genes of *Capsicum* *annuum*, *A.* *thaliana,* *S.* *lycopersicum,* and *Zea* *mays* was studied. *Capsicum* *annuum* genes are marked with a red and blue triangle. The evolutionary history was inferred using the NJ method with 1000 Bootstrap. This analysis involved 69 *ZHD* genes. Evolutionary analyses were conducted in MEGA 11 [23]

The phylogenetic tree was constructed with ZHD members of chilli (11), Arabidopsis (15), Maize (21), and *Solanum lycopersicon* (22). The result showed that these 69 ZHD genes could be divided into two groups (Group I-II), where Group I was divided into two subgroups (Ia and Ib) and Group II was divided into three subgroups (IIa, IIb, and IIc) (Fig. 4). This classification was done based on the MIF group's presence or absence. Both MIF genes from chilli and Arabidopsis are present in Clade I, consisting of *CaZHD1*, *CaZHD2*, and *CaZHD10,* along with *AtZHD5*, *AtZHD7*, *AtZHD13* and *AtZHD14* (Supplementary Table 2). Subgroup IIc had no *CaZHD* gene, while IIa contained most of the *CaZHD* genes.

### Gene chromosome mapping and collinearity analysis

**Fig. 5:**
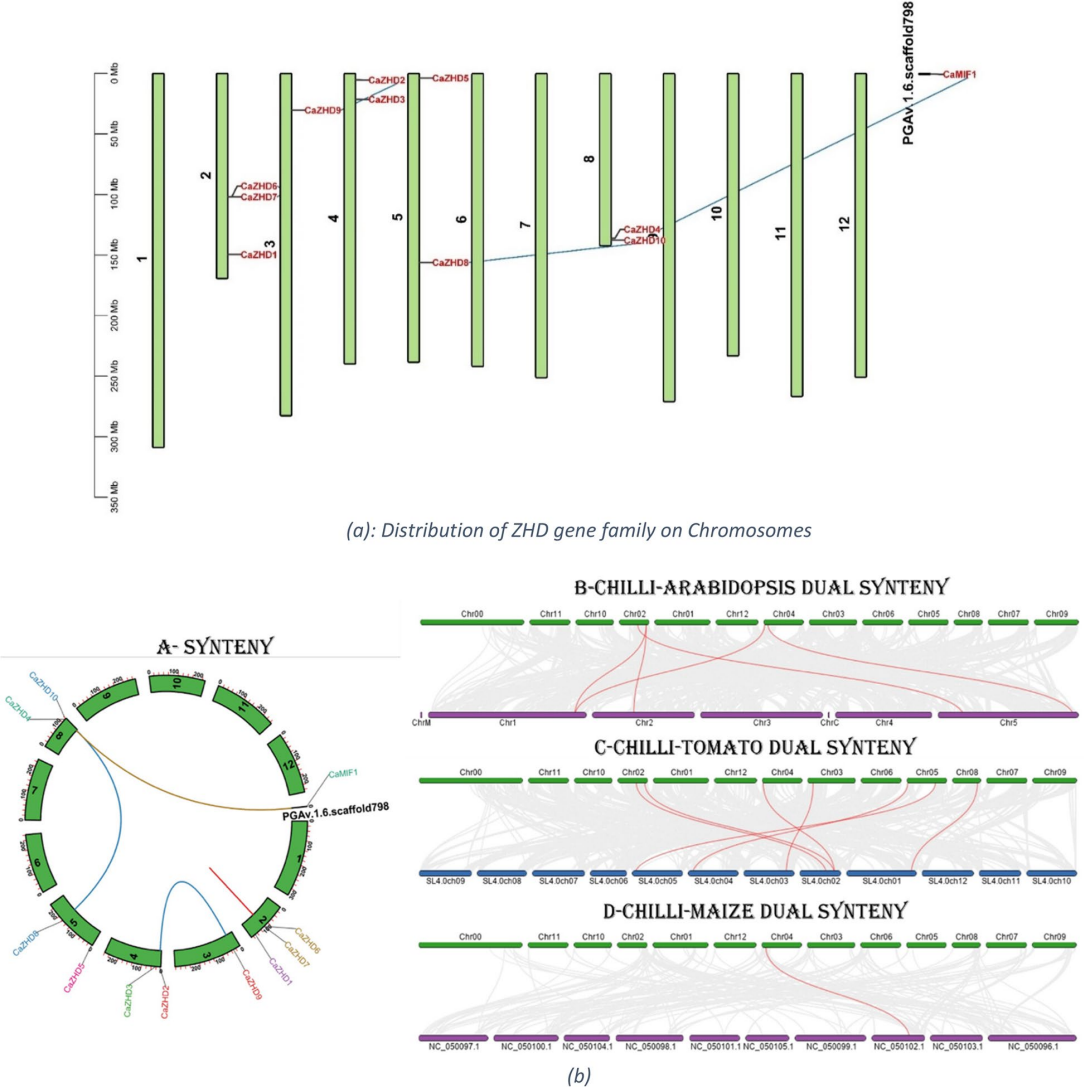
**a** Distribution of *CaZHDs* on chilli chromosomes, lines predicting the possible gene duplication on different chromosomes;** b** Genome-wide synteny analysis of *CaZHDs* and chromosomal distribution and intrachromosomal relationships of *ZHD* genes between chilli- tomato, chilli- Arabidopsis and chilli- (monocot) *Zea* *mays*. Gray lines indicate all synteny blocks in the *Capsicum* *annuum* genome, and the red lines indicate duplicated *ZHD* gene pairs. The chromosome number is indicated on the top of each chromosome, showing the dominance of segmental duplication and the rare occurrence of tandem duplication

The physical location of the chilli *CaZHDs* was identified in the chromosomes, but only *CaMIF* was located in the scaffold region of the chilli genome region (Fig. 5a). We arranged them on a pseudo-chromosome, designated as 1-12, concatenated by the unplaced scaffolds. Chromosomes 2, 3, 4, 5, and 8 contained these 11 ZHD genes, while chromosome 2 contained the highest 3 genes (Fig. 5a). The inter-relation of the genes is shown with a blue line in Fig. 5a, where Fig. 5b clarifies the location of genes in the chromosomes and scaffold. To understand the evolutionary mechanism of the ZHD gene family of chilli, we analyzed the collinear relationship between chilli and Arabidopsis, chilli and tomato, and chilli and maize (Fig. 5b). The results show that 7 pairs of collinearity genes of ZHD were between chilli and tomato, followed by chilli and Arabidopsis (5 pairs), and the least was chilli and maize (1 pair).

According to the result of the evolution analysis, the values of Ka, Ks, and Ka/Ks were obtained (Table 2). Nineteen gene pairs were identified using Tbtools. The value of Ka/Ks of each pair ranged from 0.23 to 0.29 (Ka/Ks < 1). This result indicated that all of them had undergone strong purifying selection. The CaMIF1 and CaZHD4 occurred between 213.50 MYA (Million years ago), while CaZHD6-CaZHD7 gene pair’s duplication happened most recently (around 42 MYA).

**Table 2:** Ka/Ks ratio duplicated gene pairs in chilli

Gene Id	Ka	Ks	Ka/Ks	T [33]
*CaMIF1-CaZHD4*	0.76	2.60	0.29	213.50
*CaZHD6-CaZHD7*	0.12	0.52	0.23	42.30

### Cis-acting element prediction of ZHD gene family

**Fig. 6:**
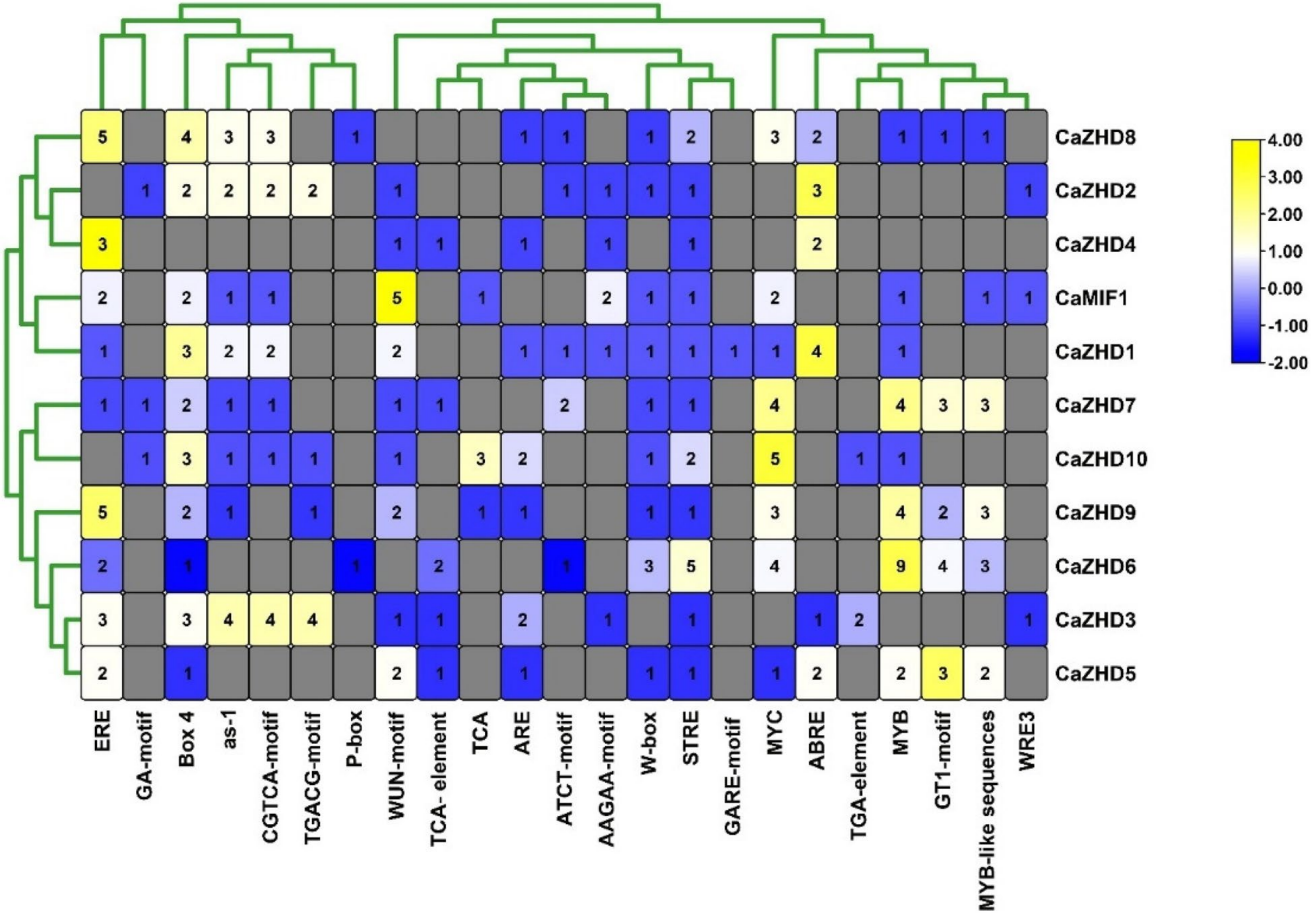
*Cis*-regulatory elements in putative *CaZHD* promoter’s process are associated with different plant developmental processes

The results of the ZHD gene family of ciss-acting element prediction of the upstream 2000 bp sequence showed various environmental and stress response elements in the ZHD gene family of chilli (Fig. 6). All CaZHDs contained light response elements STREs, while 95% of the CaZHDs contained ERE, WUN-Motif, Box-4, MYB, MYCas-1 and W-box response elements. The most common cis-elements found in our analysis were GA-motif, P-box, TCA, GARE-Motif, TGA-element, and WRE3, which were present in relatively low numbers. The remaining cis-elements accounted for approximately 50-70% of the total identified. Most of the present cis-regulatory elements were phytohormone and abiotic stress-responsible elements. For instance, ABRE cis-element regulates abscisic acid, TGA-element regulates auxin phytohormone and TCA-element salicylic acid where light-responsive STRE, AT1-motif, and Box-4 and abiotic stress-responsive MYB, MYC, ERE, and ARE present in the different *CaZHDs *(Supplementary File S1).

### Expression pattern analysis of GRF gene family

**Fig. 7:**
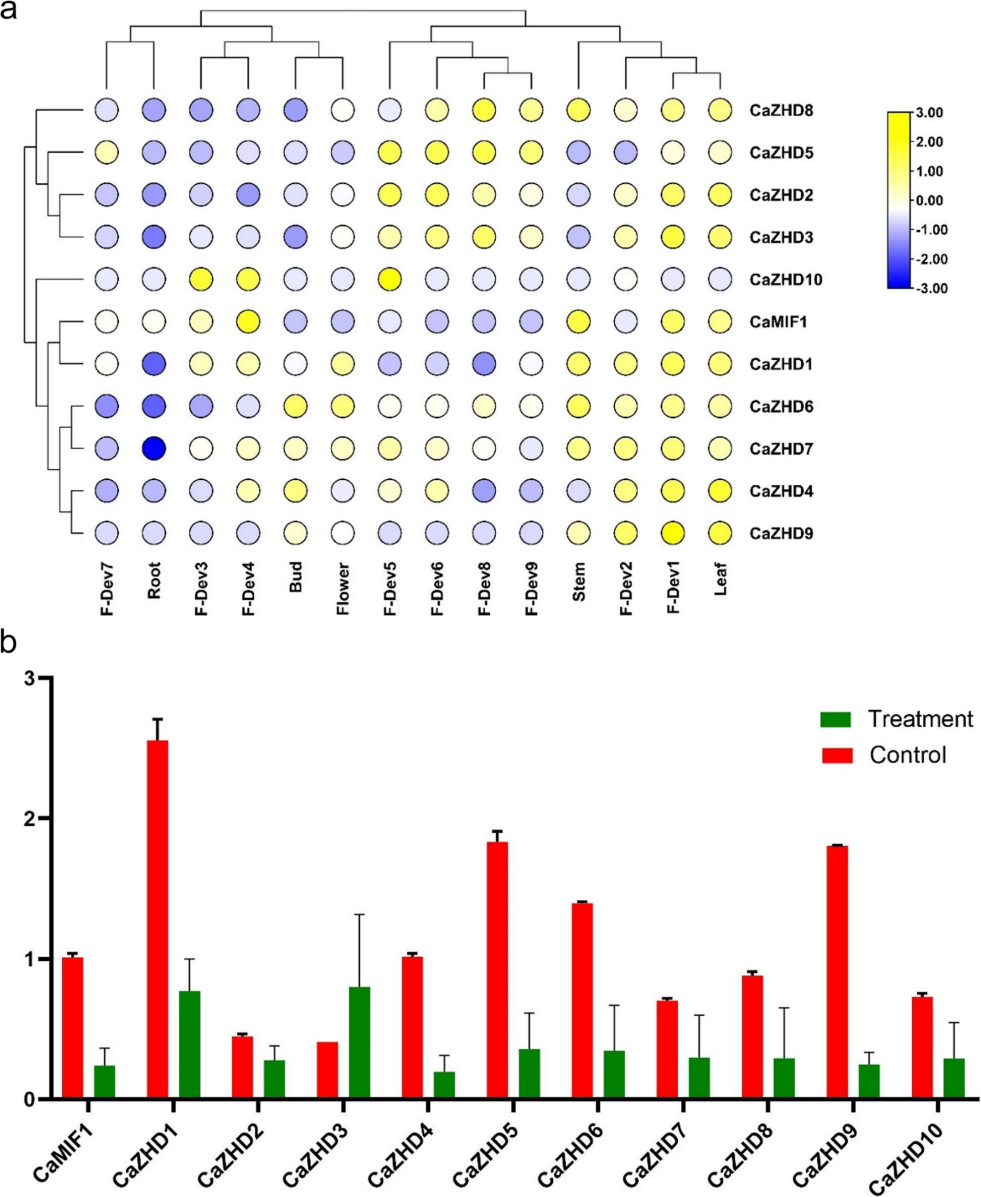
**a** The heat map shows the expression profile of the *CaZHDs* in different organs. **b** The heat map shows the expression profile of the *CaZHDs* in root organ under salt stress

Based on the transcriptome data of *Capsicum annum*, the tissue expression pattern of the ZHD gene family was analyzed, and fourteen samples, including Root, Stem, Leaf, Bud, Flower, F-Dev-1, F-Dev-2, F-Dev-3, F-Dev-4, F-Dev-5, F-Dev-6, F-Dev-7, F-Dev-8, and F-Dev-9 were selected for prediction. The result showed that the *fourteen samples expressed the CaZHDs genes differently*. The ZHD genes were expressed in Root, Stem, Leaf, Bud, Flower, F-Dev-1, F-Dev-2, F-Dev-3, F-Dev-4, F-Dev-5, F-Dev-6, F-Dev-7, F-Dev-8, and F-Dev-9, especially in the stem, leaf, flower bud, medium flower bud, and small fruit (Fig. 7a). Most the genes are expressed in leaf and fruit development stage 1 (F-Dev-1), while no gene expression in the root is sometimes expressed adversely. *CaZHD*2, 3, 5, 8 and 9 genes are responsible for the fruit maturity stage, while *CaZHD6* is highly express in bud and flower. This *CaZHD6* gene act against pathogens like nematodes resembling the *AtZHD9* gene and *CaZHD8*, and *CaZHD9* genes help ameliorate abiotic stress like drought and salinity (Table 3).

**Table 3:** Gene ontology enrichment analysis of *CaZHDs* their GO functions, Gene’s expression, Orthologs in Arabidopsis, and their functions are presented in the table

**Gene ID**	**GO Function**			**Gene expression**	**Stress**	**Ortholog in Arabidopsis**		
	**Molecular Function**	**Biological process**	**Subcellular Localization**			GeneID	Function	References
***CaMIF1***	DNA binding, DNA-binding transcription factor activity, protein binding, protein homodimerization activity, transcription cis-regulatory region binding	Embryo development by eradicating seed dormancy Transcription regulation	Nucleus	Leaf apex, Carpel, Flower, Inflorescence meristem, embryo, seed etc	Viral	*ATHB22,* *HB22,* *MEE68,* *ZHD2*	1.Seed protection from deterioration 2. Changing the impact of pathogen response	[32, 34]
***CaZHD1***	DNA, cis-regulator region and metal binding DNA binding transcription factor and protein homodimerization activity moderation;	Responsive to phytohormones except ethylene Photomorphogenesis Positive and negative regulation of meristem and transcription	Cytoplasm and nucleus	Flower, fruit, guard cell, hypocotyl, inflorescence meristem, petal, root, stem, vascular leaf	Developmental	*MIF1*	Perturbation of nitrogen associated growth and metabolism Maintaining leaf determinate growth Mediating plant development by interacting phytohormones;	[10, 33, 35, 36]
***CaZHD2***	Do	Positive transcription regulation Response to abscisic acid and abscisic acid activated signaling pathway;	Nucleus	Carpel, flower, guard cell, hypocotyl, petal, plant embryo, seed, shoot system	Developmental	*AtHB33, HB33, ZHD5*	Influenced the phloem lineage Perturbation of nitrogen associated growth and metabolism Secondary cell wall synthesis	[37–39]
***CaZHD3***	Do	Floral Meristem determination Plant type ovary development	Cytoplasm and nucleus	Mature flower	Developmental	*MIF2*	Phytohormone protein network integrated signals Floral meristem termination;	[37, 40]
***CaZHD4***	Do	Process glucosinolate metabolism Regulation transcription;	Nucleus	Carpel, flower, cotyledon, young leaf;		*ATHB21, HB21, ZFHD4, ZHD3*	Phytohormone protein network integrated signals Related to the heavy metal and drought stress tolerance	[14, 37, 41]
***CaZHD5***	Do	Seed maturation Gibberellin biosynthesis Regulation transcription;	Nucleus	Seed, carpel, young flower, young leaf	Viral infection	*ATHB25, HB25, ZFHD2, ZHD1*	Response against geminivirus and RNA viruses’ interface Increase seed longevity;	[32, 42]
***CaZHD6***	Do	Process glucosinolate metabolism Regulation transcription;	Nucleus	Flower, inflorescence, hypocotyl, dry seed, carpel, mature leaf,	Pathogen	*AtHB34, HB34, ZHD9*	Resistance against late blight and root cyst nematodes;	[43]
***CaZHD7***	Do	Transcriptional start site selection Transcriptional regulation Responsive to gibberellin and blue light Mediation of gibberellic acid signaling pathway;	Nucleus	Flower, inflorescence, hypocotyl, dry seed, carpel, mature leaf,	Developmental	*AtHB23, HB23, ZHD10*	Controlling root branching;	[44]
***CaZHD8***	Do	Responsive to water deprivation Positive regulation of transcription;	Nucleus	Seed, flower,	Abiotic stress	*ATHB29, ZFHD1, ZHD11*	Effective against drought, high salinity, and abscisic acid;	[12]
***CaZHD9***	Do	Process glucosinolate metabolism Regulation transcription;	Nucleus	Flower, inflorescence, hypocotyl, dry seed, carpel, mature leaf,	heavy metal and drought stress	*ATHB30, HB30, ZFHD3, ZHD8*	Related to the heavy metal and drought stress tolerance	[45]
***CaZHD10***	Do	Regulation transcription;	Nucleus	Flower, inflorescence, hypocotyl, dry seed, carpel, mature leaf		*ATHB32, HB32, ZHD14*	Phytohormone protein network integrated signals Evolution acting upon interactome networks	[37, 39]

### Investigating of CaZHD genes in root organ

To investigate the biological roles of CaZHD genes family by examining their expression levels in the roots of chili plants (Capsicum spp.). Our aim was to understand how these genes respond to stress conditions, specifically a high sodium chloride (NaCl) concentration of 3000 ppm from 6 weeks after transplanting [46].

Using the quantitative real-time polymerase chain reaction (qPCR) technique, we analyzed the expression patterns of 11 CaZHD genes family in the chili roots (Fig. 7b). Our findings revealed a consistent down-regulation in the expression levels of most of the ZF-HD genes examined, such as CaMIF1, CaZHD1, CaZHD2, CaZHD4, CaZHD5, CaZHD6, CaZHD7, CaZHD8, CaZHD9 and CaZHD10 under the NaCl stress conditions. However, amidst this general down-regulation trend, we made an intriguing observation. CaZHD3 displayed an up-regulation in its expression specifically in response to the NaCl stress. This differential response of CaZHD3 sets it apart from the other ZF-HD genes suggesting that it may have a distinct role in the plant's response to salt stress. Our study focused on the roots of chili plants to better understand how this crucial plant organ responds at the molecular level to the stress caused by high sodium chloride (NaCl) concentrations. By employing qPCR, we were able to assess the expression levels of the ZHD genes and highlight the potential significance of CaZHD3 as a key player in the plant's adaptive response to high NaCl concentrations. Further investigations are warranted to unravel the specific functions and regulatory mechanisms of CaZHD3 within the root system of chili plants, with the aim of enhancing salt tolerance in this important crop species.

### Annotation and ontology of the *CaZHD* gene family

**Fig. 8:**
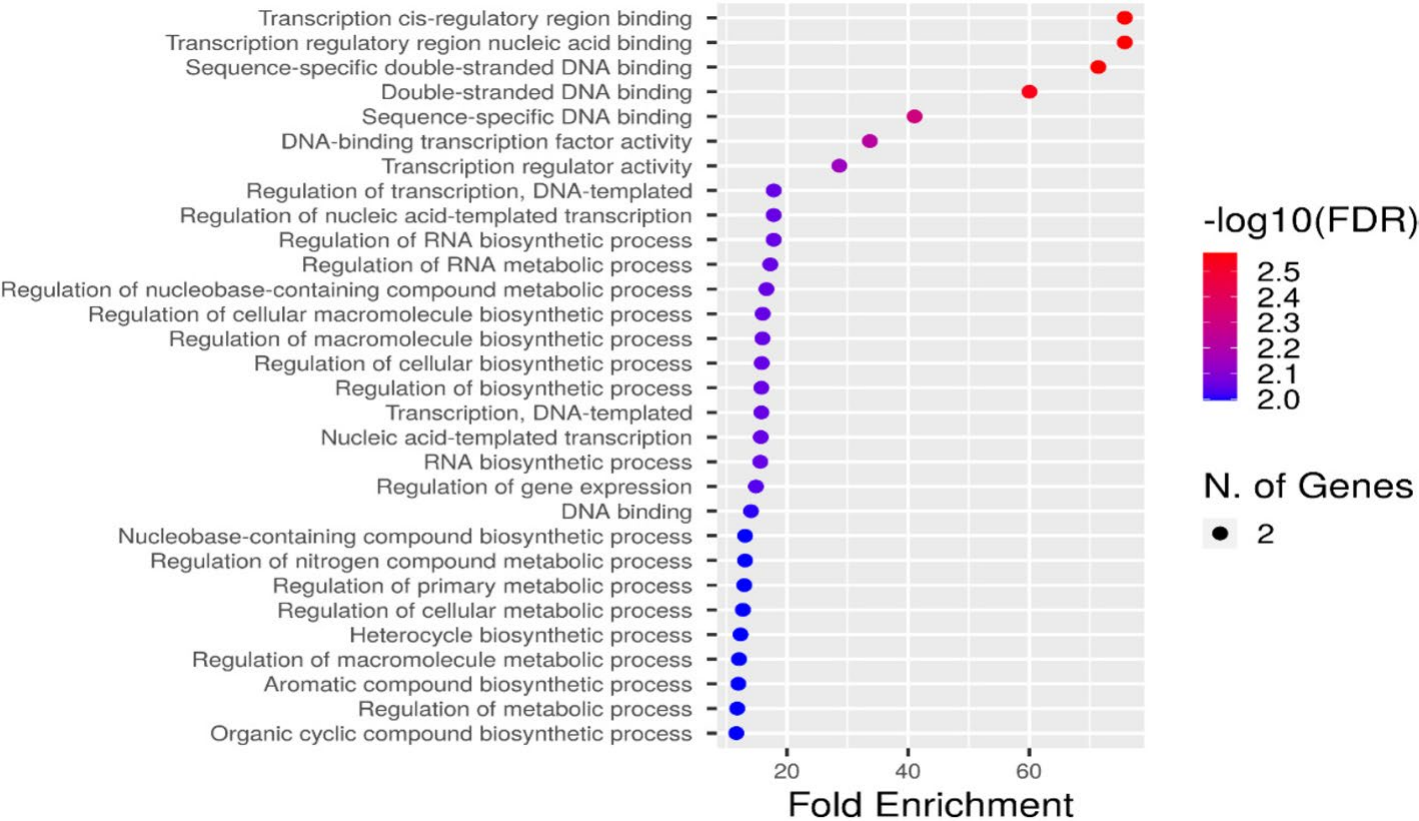
Fold Enrichment chart representing the overlapping *CaZHDs* functions. Red color dot plots represent the more no. of genes involved in that process and vice versa for small blue sizes

**Fig. 9:**
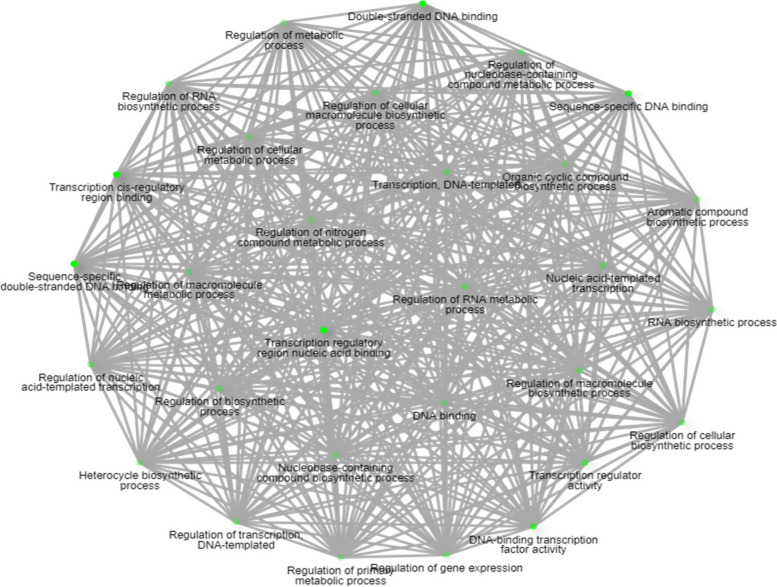
Static Network Enrichment graph showing the network of *CaZHDs* functions. Darker nodes are more significantly enriched gene sets. Bigger nodes represent larger gene sets. Thicker edges represent more overlapped genes

To have a general knowledge of the genes of the *CaZHD* gene family, the transcripts of 11 CaZHDs were annotated and categorized with gene ontology (GO). Only two of the 11 transcripts were annotated and categorized into all three primary categories, Biological Process (BP), Molecular Function (MF), and Cellular Component (CC) (Fig. 8). These two annotated genes are highly responsible for the molecular function, including transcription cis-regulatory region binding, transcription regulatory region nucleic acid binding, sequence-specific double-stranded DNA binding, double-stranded DNA binding, sequence-specific DNA binding and transcription regulatory function. These GO results of the CaZHDs were in accordance with the transcription factors' functions. The network of the molecular function depicted how they interrelated with other molecular functions and how they work (Fig. 9).

### MiRNA target site prediction and validation

MicroRNAs (miRNAs), a subclass of non-coding short RNAs with an average length of 21 nucleotides (nt), are essential for many biological processes in plants, including development, signal transduction, and responses to biotic and abiotic stress [47]. The chilli genome contains 29 conserved and 35 new miRNA families [48]. Only 9 miRNAs were found despite the prediction of 398 mature miRNA sequences against the CDSs of *CaZHDs* (Supplementary File S2, Table 5). Can-miR482a has ten target sites in *CaZHD3*, Can-miRN450a, Can-miRN450b, Can-miRN473a, Can-miRN473b, and Can-miRN473c has seven target sites in *CaZHD2*, and miRN4099 and miRN4186 have four target sites in *CaZHD4* and *CaZHD8*, respectively (Table 4).

**Table 4:** *CaZHDs* targeting Putative miRNA functions and their targeted genes

miRNA	Target gene	E-value	Length	Target-start’	Target-end’	miRNA-sequence
Can-miR482a	*CaZHD3*	5	22	515	536	CUACCAACUCCACCCAUUCCUG
Can-miRN450a	*CaZHD2*	4.5	21	813	833	CGAACUUGUCUUUUGGCACCA
Can-miRN450b	*CaZHD2*	4.5	21	813	833	CGAACUUGUCUUUUGGCACCA
Can-miRN473a	*CaZHD2*	5	22	250	271	UAUCGGUAUGAUUUUGUACACU
Can-miRN473b	*CaZHD2*	5	22	250	271	UAUCGGUAUGAUUUUGUACACU
Can-miRN473c	*CaZHD2*	5	22	250	271	UAUCGGUAUGAUUUUGUACACU
Can-miRN473d	*CaZHD2*	5	22	250	271	UAUCGGUAUGAUUUUGUACACU
Can-miRN482	*CaZHD8*	5	21	646	666	UACUUUGGGUAUUCAUAUGCU
Can-miRN482	*CaZHD4*	5	21	805	825	UACUUUGGGUAUUCAUAUGCU

### Protein–protein interaction and predicted protein structure

**Fig. 10:**
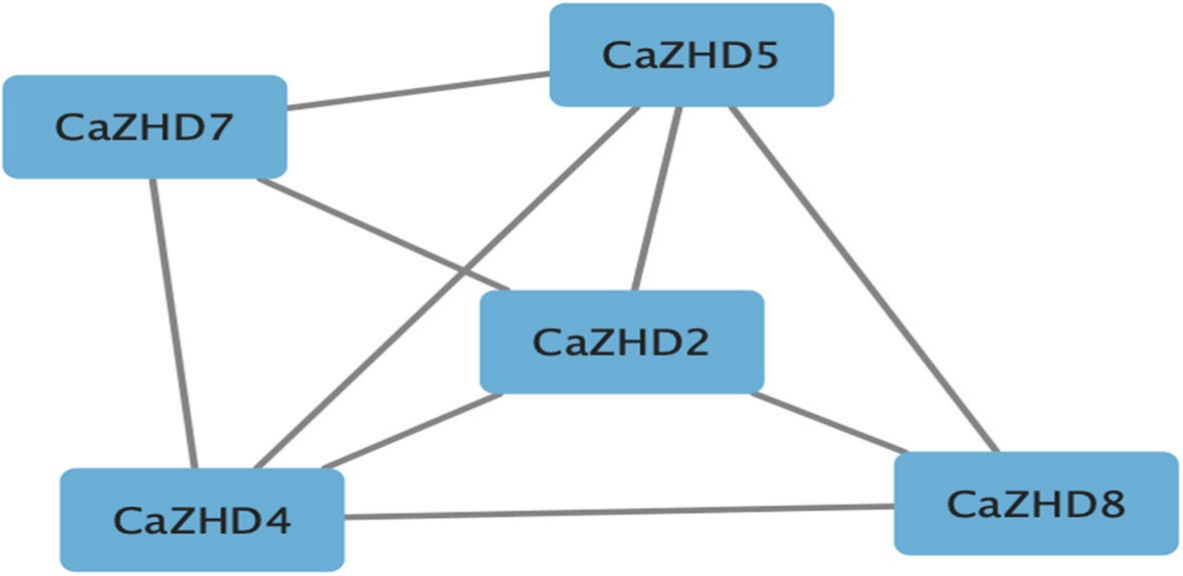
Interaction network of the ZHD proteins in *Capsicum* *annuum*. Deep ash-colored lines indicate confidence level (We selected the highest level of confidence (0.900) as the measuring unit). Protein–protein interactions (PPIs) play a crucial role in cellular functions and biological processes, including cell–cell interactions and metabolic and developmental control in all organisms

We used the STRING database to predict potential interactions among the proteins (https://string-db.org/). Only 5 proteins among 11 *CaZHD* were correlated to each at the highest level (0.900) of confidence (Fig. 10). *CaZHD2* is highly associated with other correlated genes and is speculated to play a central role in the expression and signaling. Furthermore, we illustrated protein structure with the help of online software to identify the protein structure.

## Discussion

Zinc finger Homeodomain (ZHD) transcription factors are only found in plants and play vital roles in plant growth and development and protect against biotic and abiotic stress [49]. This gene family has so far been investigated in Arabidopsis, maize, tomato, rice, cotton and wheat but not in economically beneficial hot pepper or chilli (*Capsicum annuum* L.) [4, 5, 9, 50]. Sol genomic and BLASTp studies revealed that chilli has about 11 ZHD genes. These *CaZHD* genes have persevered as most of the genes have no introns, and the most recent duplication occurred around 42 MYA (Table 2). ZHD genes were exclusively found in terrestrial plants and were more prevalent during the evolution of angiosperms [50, 51]. For the structural analysis of *CaZHD* domains, numerous evolutionary and structural studies have been carried out. Various techniques, including investigations of synteny, gene duplication, gene structure, phylogenetic trees, and motif organization were used. The size and types of conserved sections essentially present in an acceptable sequence alignment dictate the function of a gene family. Sequence comparisons revealed that motifs 1 and 4, together known as the ZF dimer (Fig. 2), are consistent with those of other plant species [50, 51], suggesting that ZHD proteins have a structure similar to those of other plants. In our tree analysis, *CaZHDs* were divided into two groups with six subgroups (Fig. 4), which aligns with other phylogenetic studies on the crops [4, 5, 52]. In subgroup IIb, most of the proteins from Arabidopsis, Tomato, and chilli were present, whereas in subgroup IIc, the *CaZHD* protein was absent, which made up most of the monocot maize protein in the tree (Fig. 4). This difference indicated that proteins from both monocots and dicots have diverged. The evolutionary insights of gene families can be extracted from the architectures of the genes belonging to the gene family [53]. Our gene structure research has revealed that many of the ZF-HD/ZHD genes lack introns (Fig. 1), a feature that has been observed in other species as well [4, 52, 54]. This suggests that the absence of introns in these genes may contribute to their ability to withstand environmental stresses, as there are no mutations that could affect their function in response to stress Therefore, according to the phylogenetic study, we can speculate that CaZHD family is an old gene family that emerged after Angiosperm split from Gymnosperm and before Dicots split from Monocots. The gene family has significantly grown since then, with the most recent gene duplication occurring between 25 and 50 MYA. Even though the feature domains of the gene family have mostly remained preserved, the gene family members have substantially diversified in terms of their nucleotide sequences, locations in the genome, and associated functionalities. The *CaZHDs* gene family was divided into two groups along with total five subgroups or subfamilies based on phylogenetic analysis and the presence or lack of conserved domains; thus, each subfamily has a unique set of conserved domains and motifs [55].

Furthermore, the functional differentiation of the *CaZHDs* genes has also been revealed at the gene expression level in different plant tissues, across developmental stages, and cultivars. For instance, almost no expression of 11 *CaZHDs* gene transcripts was found in all plant roots (Fig. 6). *CaMIF1*, *CaZHD2*, 3, 4, 8 and 9 are highly expressed in the leaf and early fruit development stage, while *CaZHD5* and *CaZHD6* were highly expressed in buds and flowers (Fig. 6). Among all eleven genes, *CaZHD5* and *CaZHD6* help to ameliorate biotic stress, and *CaZHD8* and *CaZHD9* ameliorate abiotic stress (Table 3), which resembles to the recent study [56]. From all the results taken together, we speculated that these genes, which were highly expressed in the above-ground plant parts, helped plants overcome adverse environmental conditions. These genes were also co-expressed (Fig. 10), where *CaZHD2* was centrally interrelated with others, and its main function was controlling the developmental-like phloem lineage [37, 39]. Therefore, it is clear that *CaZHD2* plays a central role in developmental and biotic and abiotic stress tolerance by controlling the other gene expression. This result was acceptable as *CaZHD2* [57–59] gene consisted of sequence-specific microRNAs, which helped to overcome biotic and abiotic stress (Table 5). During qtPCR analysis, our study found that most ZF-HD genes in the CaZHD gene family were down-regulated in the roots of chili plants under high NaCl stress, indicating their involvement in the plant's response to salt stress. However, CaZHD3 showed an intriguing up-regulation specifically in response to NaCl stress, suggesting it may have a distinct role in enhancing salt tolerance. Further investigations are needed to uncover the precise functions and regulatory mechanisms of CaZHD3 in the root system, contributing to a comprehensive understanding of salt stress adaptation in chili plants [55, 60–62]. Though some of the miRNA functions are still not found, we need to explore to know the more complex mechanisms. microRNAs are important plant regulators that regulate almost every biological process, from growth and development to combating pathogens and maintaining proper internal conditions [63, 64]. The molecular mechanism of the 11 *CaZHDs* was predicted through GO and network analysis. Only 2 genes showed molecular functions, and their molecular functions were highly correlated with each and made an intriguing network (Figs. 8 and 9). In addition, as the *CaZHD* genes' cis-regulatory elements control the phytohormonal signaling and abiotic stress tolerance mechanism, we can predict that these genes' microRNAs are also correlated with them as they are sequence-specific [62, 67, 68].

**Table 5:** *CaZHDs* targeting Putative miRNA functions along with their targeted genes

miRNA	Target gene	Function	References
Can-miR482a	*CaZHD3*	Regulates NBS-LRR defense genes amid pathogen infection	[65]
Can-miRN450a	*CaZHD2*	Acting as a tumor suppressor in ovarian cancer cells	[66]
Can-miRN450b	*CaZHD2*	Treating glioblastoma (GBM) disease in human	[50]
Can-miRN473a	*CaZHD2*	Not reported	
Can-miRN473b	*CaZHD2*	Not reported	
Can-miRN473c	*CaZHD2*	Not reported	
Can-miRN473d	*CaZHD2*	Not reported	
Can-miRN482	*CaZHD8*	Embryo development Targeting mRNAs for NBS-LRR disease resistance;	[48, 65]
Can-miRN482	*CaZHD4*	Embryo development Targeting mRNAs for NBS-LRR disease resistance;	[48, 65]

## Conclusions

It has been determined that the CaZHD gene family in hot pepper (*Capsicum annuum* L.) consists of 11 genes. Before the Monocots and Dicots division and following the separation between Angiosperm and Gymnosperm, the *CaZHD* gene family most likely developed. The family was divided into two subfamilies, and these subfamilies differed substantially in terms of chromosomal position, nucleotide sequence, and GO annotation and categorization. Even while the expression of the genes in the family varied significantly in four-year-old plant tissues, four-year-old plant roots, and four-year-old plant roots from various cultivars, the functional relationships between the genes in the family persisted. Two *CaZHDs* were crucial in the chilli plant's biotic stress, and two were confirmed to control the plant's response to abiotic stress.

### Supplementary Information

**Additional file 1.**

**Additional file 2.**

**Additional file 3: Supplementary Table 1.** Information regarding Motifs.

**Additional file 4: Supplementary Table 2.** Chili’s *ZHD *gene family distribution among groups based on phylogenetic analysis.

## Data Availability

All data generated or analysed during this study are included in this published article and its supplementary information files.
