# Supplementary material for: In silico and computational analysis of zinc finger motif-associated homeodomain (ZF-HD) family genes in chilli (Capsicum annuum L)
Source: BMC Genomics. 2023 Oct 11;24:603. doi: 10.1186/s12864-023-09682-x (PMC10566081; doi:10.1186/s12864-023-09682-x)
Supplement: Supplementary file 3 — Additional file 3: Supplementary Table 1. Information regarding Motifs. [file 12864_2023_9682_MOESM3_ESM.docx]

**Supplementary Tables**

**Supplementary Table 1. Information regarding Motifs**

| Serial No. | Motif sequence | Width |
| --- | --- | --- |
| 1 | VRYKECLKNHAASJGGHAVDGCGEFMPSG | 29 |
| 2 | FRTKFTQEQKEKMLEFAERLGWKIQKQDEDEVQE | 34 |
| 3 | GTPESLKCAACGCHRNFHRKE | 21 |
| 4 | FCREIGVKRQVLKVWMHNNKN | 21 |
| 5 | TTTTTTTTPKSPEPESETPTRIQPAKPISFSNGIIKRHHHH | 41 |
| 6 | PPPPTAAIEYQPHHRHHPPPPPPPPLPRS | 29 |
| 7 | PNSASPPPISSSYMLLALSGGNNGENN | 27 |
| 8 | TFNRRDIAGNEIRQIDNGGGNHTPILAGEINNHNNGHHGVGGGGELHQSV | 50 |
| 9 | HHHHHH | 6 |
| 10 | SGGGGGGFESDSGGA | 15 |
| 11 | RKLMFNHKKIKSPLPQQMIMPIGV | 24 |
| 12 | GGNTNGSSSS | 10 |
| 13 | YLHHPPPPHQPPLALPSTS | 19 |
| 14 | MELASQEEDM | 10 |
| 15 | MMDMTP | 6 |
| 16 | LASFSDLNFSA | 11 |
| 17 | NNNVNGFCIVSR | 12 |
| 18 | DNNNBS | 6 |
| 19 | EVMGWRWAKW | 10 |
| 20 | PCQLQPLAPPPNLJLSLSSGFFGPSDQ | 27 |
