# Supplementary material for: In silico and computational analysis of zinc finger motif-associated homeodomain (ZF-HD) family genes in chilli (Capsicum annuum L)
Source: BMC Genomics. 2023 Oct 11;24:603. doi: 10.1186/s12864-023-09682-x (PMC10566081; doi:10.1186/s12864-023-09682-x)
Supplement: Supplementary file 4 — Additional file 4: Supplementary Table 2. Chili’s ZHD gene family distribution among groups based on phylogenetic analysis. [file 12864_2023_9682_MOESM4_ESM.docx]

**Supplementary Table 2.** Chili’s *ZHD* gene family distribution among groups based on phylogenetic analysis.

| **Groups** | **Subgroups** | **Chilli (*Capsicum annuum*)** | | **Arabidopsis** | | **Tomato** | | **Maize** | |
| --- | --- | --- | --- | --- | --- | --- | --- | --- | --- |
|  |  | **Number** | **Gene Name** | **Number** | **Gene Name** | **Number** | **Gene Name** | **Number** | **Gene Name** |
| I | Ia | 2 | *CaZHD1; CaZHD10* | 1 | *AtMIF1* | 2 | *SL-ZH14; SL-ZH10* | 5 | *ZmZHD1, ZmZHD4, ZmZHD8; ZmZHD13; ZmZHD21* |
|  | Ib | 2 | *CaMIF1; CaZHD2* | 4 | *AtZHD5; AtZHD7; AtZHD13; AtZHD14* | 9 | *SL-ZH1; SL-ZH3; SL-ZH4; Sl-ZH9; SL-ZH12; SL-ZH17; SL-ZH19; SL-ZH20; SL-ZH22* | 7 | *ZmZHD2; ZmZHD9; ZmZHD10; ZmZHD12; ZmZHD17; ZmZHD18; ZmZHD20* |
| II | IIa | 6 | *CaZHD4; CaZHD5; CaZHD6; CaZHD7; CaZHD8; CaZHD9* | 6 | *AtZHD3; AtZHD4; AtZHD6; AtZHD8; AtZHD9; AtZHD10* | 9 | *SL-ZH2; SL-ZH5; SL-ZH6; SL-ZH7; SL-ZH8; SL-ZH13; SL-ZH15, SL-ZH18; SL-ZH21* | 2 | *ZmZHD14; ZmZHD19* |
|  | IIb | 1 | *CaZHD3* | 3 | *AtZHD1; AtZHD2; AtZHD12* | 1 | *SL-ZH16* | 3 | *ZmZHD3; ZmZHD15; ZmZHD16* |
|  | IIc | 0 |  | 1 | *AtZHD11* | 1 | *SL-ZH11* | 4 | *ZmZHD5; ZmZHD6; ZmZHD7; ZmZHD11* |
